# Disarming Multidrug‐Resistant Pathogens: Sodium Dodecyl Sulfate–Induced Plasmid Curing for the Reversal of Resistance and Virulence in *Escherichia coli*

**DOI:** 10.1155/ijm/5568321

**Published:** 2026-06-19

**Authors:** Alyaa I. Eliwa, Hagar S. M. Nassar, Maha M. Eldahshan

**Affiliations:** ^1^ Department of Medical Microbiology and Immunology, Faculty of Medicine, Menoufia University, Shebin Elkom, Egypt, menofia.edu.eg; ^2^ Department of Clinical Pathology, Faculty of Medicine, Menoufia University, Shebin Elkom, Egypt, menofia.edu.eg

**Keywords:** antimicrobial resistance, *Escherichia coli*, multidrug resistance, plasmid curing, sodium dodecyl sulfate, virulence

## Abstract

**Introduction:**

The global escalation of multidrug‐resistant (*MDR*) *Escherichia coli* poses a serious clinical challenge, largely driven by plasmid‐mediated dissemination of antimicrobial resistance and virulence determinants. This study is aimed at evaluating the effectiveness of sodium dodecyl sulfate (SDS)–induced plasmid curing in reversing antimicrobial resistance and virulence phenotypes in clinical MDR *E. coli* isolates.

**Methods:**

A total of 485 clinical specimens were collected from hospitalized patients, yielding 190 *E. coli* isolates. All *E. coli* isolates (*n* = 190) were subjected to antimicrobial susceptibility testing and virulence phenotyping; of these, 63 were identified as MDR and subjected to plasmid profiling. A subset of 50 highly resistant isolates, defined as those resistant to ≥ 5 antimicrobial agents, was subsequently selected for SDS‐mediated curing, followed by postcuring molecular and phenotypic reassessment.

**Results:**

*E. coli* was isolated from 39.2% of specimens, with 33.2% classified as MDR. SDS curing achieved 84.0% plasmid elimination, resulting in marked reductions in resistance to *β*‐lactams and fluoroquinolones (≈45%–55%) and significant attenuation of virulence traits, including hemolysin production (53.3%) and motility (62.8%). All paired pre‐/postcuring comparisons reached statistical significance (McNemar′s test, *p* < 0.001 for the principal *β*‐lactam, fluoroquinolone, and virulence outcomes). A strong molecular–phenotypic concordance (*r*_*S*_ = 0.83) was observed.

**Conclusions:**

SDS‐induced plasmid curing effectively reverses resistance and virulence in clinical MDR *E. coli*, underscoring the central role of plasmids in shaping pathogenicity and supporting plasmid curing as an experimental laboratory tool rather than a clinically applicable intervention for dissecting plasmid‐borne resistance and virulence determinants and informing future antimicrobial stewardship‐aligned strategies.

## 1. Introduction

*Escherichia coli*, a ubiquitous commensal member of the *Enterobacteriaceae* family, is also a leading pathogen responsible for infections ranging from urinary tract and gastrointestinal diseases to life‐threatening bloodstream infections. Its clinical relevance is amplified by the global surge in multidrug resistance (MDR), often mediated by mobile genetic elements such as plasmids carrying antibiotic resistance genes (ARGs) and virulence determinants [1, 2]. Plasmid‐driven horizontal gene transfer enables the rapid dissemination of resistance phenotypes across bacterial populations, compounding the antimicrobial resistance (AMR) crisis and critically limiting therapeutic options [3, 4].

Of particular concern are MDR *E. coli* strains harboring conjugative plasmids encoding extended‐spectrum *β*‐lactamases (ESBLs), carbapenemases, aminoglycoside‐modifying enzymes, and quinolone resistance determinants, which significantly undermine frontline treatments [5]. In parallel, plasmid‐encoded virulence traits such as hemolysins and factors enhancing motility and colonization have been reported to exacerbate disease severity and clinical outcomes, although the contribution of plasmid‐borne versus chromosomal determinants is strain‐dependent and varies between phylogroups, underscoring the dual role of plasmids in resistance and pathogenicity [3].

Traditional antimicrobial therapies are increasingly inadequate against such dual threats, as resistance mechanisms may be encoded both chromosomally and on plasmids. Consequently, novel strategies targeting plasmids directly are gaining attention. Plasmid curing, which reduces or eliminates plasmid DNA without killing the bacterial host, holds promise as it simultaneously attenuates resistance and virulence while minimizing selective pressures that drive new resistance evolution [6, 7]. A variety of approaches, including chemical agents, elevated growth temperature, detergents, and CRISPR–Cas–based interventions, have been explored for plasmid curing over the past decades [8, 9]. Among these, sodium dodecyl sulfate (SDS) remains the most widely used reference curing agent because of its low cost, simple workflow, broad applicability across gram‐negative bacteria, and its predominant action on plasmid replication and segregation rather than on host genomic DNA [6, 8], in contrast to intercalating agents such as acridine orange or ethidium bromide that may also damage chromosomal DNA [6], and CRISPR–Cas tools that require sequence‐specific design and delivery vehicles not yet adapted for routine clinical‐isolate workflows [3].

Among chemical agents, SDS, a widely used anionic detergent, has been shown to effectively cure plasmids by disrupting bacterial membranes, altering plasmid topology, and interfering with replication and segregation processes [6, 10]. SDS‐mediated curing has reversed resistance phenotypes and attenuated virulence in several bacterial pathogens, including *Escherichia*, *Klebsiella*, and *Pseudomonas* species [5, 11, 12].

Despite these promising reports, comprehensive studies focusing on SDS plasmid curing in clinical MDR *E. coli* isolates remain limited. Although classical SDS plasmid‐curing assays have been described since [8] and applied sporadically to environmental and clinical isolates, few contemporary studies have systematically integrated plasmid profiling, time‐course curing kinetics, and parallel phenotypic reassessment of both AMR and virulence in the same clinical MDR *E. coli* cohort. The novelty of the present work lies in this integrated framework, applied to a substantial clinical MDR cohort and including stratified molecular–phenotypic outcomes. In particular, few investigations have correlated plasmid elimination with phenotypic reversion of resistance and virulence, or provided detailed molecular profiles postcuring. Bridging this knowledge gap is critical for assessing the conceptual translational potential of plasmid‐targeted interventions within antimicrobial‐resistance research frameworks.

This study is aimed at evaluating the effectiveness of SDS‐induced plasmid curing in MDR *E. coli* clinical isolates collected from diverse infection sites. By systematically comparing antimicrobial susceptibility, plasmid DNA profiles, and virulence‐associated phenotypes before and after curing, we sought to determine the extent to which MDR and virulence are plasmid‐borne. The findings provide mechanistic insights into the feasibility of plasmid curing as an experimental laboratory tool for dissecting resistance reversal rather than as a directly applicable therapeutic intervention.

## 2. Materials and Methods

### 2.1. Study Design and Ethical Considerations

This prospective study was conducted in the Department of Medical Microbiology and Immunology, Faculty of Medicine, Menoufia University, Egypt, between 2025 and 2026. A total of 485 clinical specimens were collected from hospitalized patients admitted to multiple wards and intensive care units (ICUs) with clinically suspected infections. The study protocol was reviewed and approved by the Institutional Ethics Committee of Menoufia University (IRB Reference Number: 2026CPATH12). Written informed consent was obtained from all participants or their legal guardians in accordance with the Declaration of Helsinki.

### 2.2. Clinical Specimen Collection and *E. coli* Isolation

Clinical specimens were aseptically collected according to the suspected site of infection and included sputum, wound swabs, urethral swabs, catheterized and midstream urine, nasal swabs, high vaginal swab specimens, blood, and stool. Samples were transported to the microbiology laboratory under refrigerated conditions (4°C) and processed within 2 h of collection. Specimens were cultured on standard bacteriological media (Oxoid, England) under appropriate conditions, and presumptive *E. coli* isolates were identified using standard phenotypic and biochemical methods. Confirmed isolates were preserved in 20% glycerol at −80°C and revived prior to experimentation by subculture on Luria–Bertani (LB) agar at 37°C for 18–24 h to ensure purity and viability.

### 2.3. Antimicrobial Susceptibility Testing and MDR Determination

Antimicrobial susceptibility testing was performed using the Kirby–Bauer disk diffusion method on Mueller–Hinton agar according to CLSI 2025 [13] interpretive criteria, employing representative antibiotics from penicillins, cephalosporins, carbapenems, aminoglycosides, tetracyclines, fluoroquinolones, and trimethoprim–sulfamethoxazole; nitrofurantoin was tested only for urinary isolates in accordance with its clinical indication. Macrolides were not included in the susceptibility analysis, multiple antibiotic resistance index (MARI), or multidrug resistance index (MDRI) calculations because *E. coli* exhibits intrinsic resistance to macrolides via outer‐membrane permeability barriers; their inclusion would artefactually inflate resistance indices and obscure plasmid‐mediated changes after curing. Erythromycin appears in the descriptive MDR profiles in Table 1 only as a phenotypic profile descriptor and was not used in any quantitative resistance index.

**Table 1:** Representative multidrug resistance (MDR) profiles among *E. coli* isolates (n = 63).

MDR profile	*n* (%)	MDRI	Interpretation
LBC–AZN–ERY–CD–CIP–P–OFX	12 (19.0)	0.70	Broad‐spectrum resistance
CIP–LBC–ERY–P–CD–OFX	10 (15.9)	0.60	Common mobile resistance pattern
LBC–P–GX–CN–OFX–AZN–ERY–CRO	8 (12.7)	0.70	Includes third‐generation cephalosporins
CRO–ERY–P–LBC–OFX–AZN–AUG–ZEM	7 (11.1)	0.75	Extended *β*‐lactam resistance
ZEM–LBC–CIP–CRO–P–CN	6 (9.5)	0.65	Fluoroquinolone–cephalosporin coresistance
AZN–LBC–ERY–P–CIP–CRO	5 (7.9)	0.68	Multiclass resistance
Other less frequent profiles	15 (23.8)	0.30–0.60	Lower‐complexity MDR

*Note:* MDRI, multidrug resistance index = number of antimicrobial classes to which the isolate is resistant/total number of antimicrobial classes tested. ERY appears as a phenotypic profile descriptor only and was not used in MARI/MDRI calculations because of intrinsic *E. coli* macrolide resistance.

Abbreviations: AZN, aztreonam; CD, clindamycin/lincosamide descriptor; CIP, ciprofloxacin; CN, gentamicin; CRO, ceftriaxone; ERY, erythromycin (descriptor only); GX, gatifloxacin; LBC, levofloxacin/*β*‐lactam combination descriptor; OFX, ofloxacin; P, penicillin; ZEM, cefepime/cefixime descriptor.

MDR was defined as acquired nonsusceptibility to at least one agent in three or more antimicrobial categories, in accordance with the standardized international definition proposed by Magiorakos et al. [14]. To quantify resistance intensity, the MARI was calculated for each isolate as follows:

MARI= number of antibiotics to which the isolate was resistanttotal number of antibiotics tested

Selection criteria for the 63 MDR isolates were strictly the [14] definition applied to all 190 *E. coli* isolates; no further filtering (clinical site, ward, or patient demographics) was applied. From these 63, a subset of 50 isolates was selected for SDS‐induced plasmid curing using a single, prespecified criterion: phenotypic resistance to ≥ 5 antimicrobial agents across at least three antimicrobial classes, in order to enrich for highly resistant phenotypes most likely to harbor plasmid‐borne resistance determinants. The remaining 13 MDR isolates resistant to < 5 agents were excluded from the curing experiment but were retained in the descriptive MDR analyses (Tables 2 and 1 and Table S1). In addition to the MARI, a MDRI was calculated to reflect resistance complexity at the antibiotic class level. MDRI was defined as the ratio of the number of antimicrobial classes to which an isolate was resistant to the total number of antimicrobial classes tested, and was calculated as follows: MDRI = (number of antimicrobial classes showing resistance)/(total number of antimicrobial classes tested). This index provides an estimate of resistance breadth across antimicrobial categories rather than individual agents and has been used to describe the complexity of MDR in clinical bacterial isolates [15, 16].

**Table 2:** Antibiotic resistance, susceptibility, and coresistance patterns among MDR *E. coli* isolates (*n* = 63).

Antibiotic	Resistant, *n* (%)	Susceptible, *n* (%)	Coresistance indicator
Ampicillin	57 (90.5)	6 (9.5)	≥ 1 *β*‐lactam
Amoxicillin–clavulanate	54 (85.7)	9 (14.3)	—
Piperacillin–tazobactam	43 (68.3)	20 (31.7)	—
Ceftriaxone	56 (88.9)	7 (11.1)	≥ 1 third‐generation cephalosporin
Ceftazidime	52 (82.5)	11 (17.5)	—
Cefepime	47 (74.6)	16 (25.4)	—
Imipenem	15 (23.8)	48 (76.2)	≥ 1 carbapenem
Meropenem	14 (22.2)	49 (77.8)	—
Gentamicin	44 (69.8)	19 (30.2)	—
Amikacin	27 (42.9)	36 (57.1)	—
Ciprofloxacin	53 (84.1)	10 (15.9)	≥ 1 fluoroquinolone
Levofloxacin	50 (79.4)	13 (20.6)	—
Tetracycline	47 (74.6)	16 (25.4)	—
Trimethoprim–sulfamethoxazole	39 (61.9)	24 (38.1)	—
Nitrofurantoin^a^	17 (62.9)	10 (37.1)	Urinary MDR only (*n* = 27)

*Note:* Macrolides were excluded from the susceptibility analysis owing to intrinsic *E. coli* resistance. Coresistance indicators denote at least one resistant agent within the corresponding class. Susceptibility interpreted per CLSI M100 (2025).

Abbreviation: MDR, multidrug‐resistant.

^a^Nitrofurantoin susceptibility was tested only for urinary MDR isolates (*n* = 27).

### 2.4. Baseline Phenotypic Characterization of Resistance and Virulence

Prior to plasmid curing, MDR isolates underwent baseline phenotypic characterization, including confirmation of antimicrobial susceptibility patterns by disk diffusion [13] and assessment of virulence‐associated traits.

#### 2.4.1. Hemolysin Production

Isolates were streaked onto 5% sheep blood agar and incubated at 37°C for 24 h. The presence of a clear zone of complete (*β*) hemolysis around colonies was interpreted as positive hemolysin activity.

#### 2.4.2. Motility Testing

Motility was evaluated using semisolid motility medium (0.3% agar). Isolates were stab‐inoculated and incubated at 37°C for 24–48 h. Diffuse growth radiating from the stab line indicated a motile phenotype.

### 2.5. SDS‐Induced Plasmid Curing

Plasmid curing experiments were performed on a subset of 50 MDR *E. coli* clinical isolates demonstrating resistance to five or more antibiotics, to enrich for highly resistant phenotypes likely associated with plasmid‐borne determinants. SDS curing procedures were conducted using approaches adapted from established protocols [8, 12].

A 10% (*w*/*v*) SDS stock solution was prepared in distilled water, sterilized by 0.22‐*μ*m filtration, and added to nutrient broth to achieve a final working concentration of 10% (*w*/*v*) in the curing cultures. This concentration was selected on the basis of preliminary growth‐inhibition optimization experiments performed on five randomly selected MDR isolates, in which serial concentrations (1%, 2.5%, 5%, 7.5%, and 10% *w*/*v*) were tested; 10% (*w*/*v*) yielded subinhibitory growth permitting curing without complete bactericidal effect, consistent with previously published SDS‐curing protocols for Enterobacteriaceae [8, 12]. Overnight cultures of MDR isolates were diluted to approximately 10^3^ CFU/mL in SDS‐supplemented nutrient broth and incubated at 37°C with shaking (150 rpm) for 24–48 h. Parallel SDS‐free cultures were included as passaging controls and subjected to identical subculturing and phenotypic reassessment. All curing experiments were performed in biological triplicate with technical duplicates per time point. Isolates exhibiting complete growth inhibition under SDS exposure were excluded to ensure analysis of curing outcomes only in viable isolates.

Following incubation, cultures were serially diluted and plated on antibiotic‐free LB agar to obtain isolated colonies. Putative cured variants were screened by replica plating: Each colony from antibiotic‐free LB was transferred in parallel onto LB agar with and without amoxicillin (25 *μ*g/mL) using a sterile velvet replicator and incubated at 37°C for 24 h. Colonies growing on antibiotic‐free plates but failing to grow on amoxicillin‐containing plates were considered presumptive susceptibility‐reverted derivatives and were subsequently confirmed by plasmid profiling. These isolates were stabilized by subculturing in antibiotic‐free nutrient broth at 37°C for 24 h prior to molecular confirmation [8].

### 2.6. Plasmid DNA Extraction and Agarose Gel Electrophoresis

Plasmid DNA was extracted from *E. coli* isolates before and after SDS curing using the QIAprep Spin Miniprep Kit (QIAGEN, Germany) according to the manufacturer′s instructions, with minor optimization. Briefly, isolates were cultured in Mueller–Hinton broth at 37°C for 18–24 h. Cells were harvested by centrifugation (10,000 × g, 10 min) and lysed using Tris–EDTA buffer with sodium acetate. Plasmid DNA was precipitated with cold 70% ethanol, washed, air‐dried, resuspended in TE buffer, and stored at 4°C. Plasmid DNA was analyzed by 1% agarose gel electrophoresis in TAE buffer, stained with ethidium bromide (100 V, 30 min), and visualized under UV transillumination. Curing outcomes were categorized as follows: complete curing, defined as the disappearance of all detectable plasmid bands compared with the parental isolate; partial curing, defined as a reduction in the number of plasmid bands; and no curing, defined as an unchanged plasmid profile. Putative cured derivatives were further stabilized by three sequential passages in antibiotic‐free media before reassessment [6, 17, 18].

### 2.7. Determination of the Molecular Weight of the *E. coli* Plasmids

Plasmid molecular weight was determined by comparing the migration distances of plasmid DNA bands with those of a standard DNA molecular weight marker, as described by Aladejana et al. [17] and Okoye et al. [18]. A GelPilot 1‐kb DNA ladder (1000–10,000 bp; QIAGEN, Germany) was used as the reference standard. Following electrophoresis, plasmid DNA bands were visualized under UV transillumination, and plasmid profiles were analyzed and compared among isolates before and after curing. Because the reference ladder spanned 1–10 kb, accurate plasmid size estimation was restricted to this interval. Larger plasmids commonly associated with ESBL/AmpC/carbapenemase carriage (e.g., IncF, IncN, IncL/M, and IncHI2; typically 50–250 kb) lie outside the resolvable range of the ladder used and may therefore have been undetected or misclassified. This is a methodological limitation and the plasmid sizes reported here should not be overgeneralized to the full plasmid content of these isolates.

### 2.8. Time‐Course Kinetics of SDS Curing

To assess curing kinetics, 50 MDR *E. coli* isolates exposed to SDS (10% *w*/*v*) were sampled at 0, 6, 12, 24, and 48 h. All time‐course experiments were performed in biological triplicate with technical duplicates. At each time point, aliquots were serially diluted and plated on antibiotic‐free LB agar, and representative colonies were screened for resistance loss by replica plating onto LB agar with and without amoxicillin (25 *μ*g/mL). Isolates were considered resistant if at least one colony grew on amoxicillin‐containing plates; otherwise, they were classified as susceptibility‐reverted. Plasmid retention was confirmed by plasmid extraction and agarose gel electrophoresis from representative colonies, and expressed as the percentage of isolates retaining detectable plasmid bands relative to the total tested (*n* = 50). Resistance reduction (%) was calculated as the decrease in resistant isolates relative to baseline (0 h) [6, 8].

### 2.9. Postcuring Assessment of Resistance and Virulence

Following molecular confirmation of plasmid loss by agarose gel electrophoresis, putative postcuring isolates were reassessed to determine phenotypic changes associated with plasmid elimination. Antibiotic susceptibility testing was repeated using the Kirby–Bauer disk diffusion method to evaluate reductions or loss of resistance patterns following SDS exposure. In addition, hemolysin production and motility assays were repeated using the same baseline procedures to assess attenuation of virulence‐associated phenotypes in cured derivatives.

### 2.10. Determination of Plasmid Curing Efficiency

Plasmid curing efficiency was calculated as follows: Curing efficiency (*%*) = [(number of isolates showing plasmid loss and resistance loss) / (total number of isolates tested)] × 100.

Phenotypic reversion rates were calculated similarly as the proportion of isolates showing complete or partial susceptibility/virulence reversion after curing.

### 2.11. Statistical Analysis

Statistical analysis was performed using SPSS Version 20 (IBM Corp., Armonk, New York, United States). Categorical variables were expressed as frequencies and percentages, whereas continuous variables were summarized as means and standard deviations where applicable. For paired pre‐/postcuring categorical outcomes within the same isolate (resistance status to each antibiotic and presence/absence of each virulence trait), McNemar′s test for paired binary data was applied (Tables 3 and 4). Exact two‐sided *p* values are reported, and 95% confidence intervals (CIs) for differences in paired proportions were estimated using the Newcombe–Wilson method. Chi‐square tests (or Fisher′s exact test where expected counts < 5) were used for comparisons between independent groups. To address reviewer comments regarding correlation analysis, the molecular–phenotypic association in Table 5 was tested using Spearman′s rank correlation after coding both molecular and phenotypic outcomes as ordinal variables (no loss = 0; partial loss = 1; complete loss = 2), which was confirmed by an independent ordinal trend test (Cochran–Armitage). Sensitivity analyses for discordant pairs were additionally explored using a Bayesian beta‐binomial framework with noninformative priors (Beta[1]) to estimate posterior probabilities of true reversion; results were concordant with the frequentist analyses and are reported in the Results. All statistical analyses were two‐tailed, and a *p* value < 0.05 was considered statistically significant.

**Table 3:** Reduction in antimicrobial resistance after SDS‐induced plasmid curing (n = 50).

Antibiotic	Precuring resistant, *n* (%)	Postcuring resistant, *n* (%)	Absolute change (%)	Reduction rate (%)	*p*
Ampicillin	46 (92.0)	21 (42.0)	**50.0**	54.3	< 0.001
Amoxicillin–clavulanate	43 (86.0)	23 (46.0)	**40.0**	46.5	< 0.001
Piperacillin–tazobactam	34 (68.0)	18 (36.0)	**32.0**	40.0	< 0.05
Ceftriaxone	45 (90.0)	22 (44.0)	**46.0**	51.1	< 0.001
Ceftazidime	43 (86.0)	24 (48.0)	**38.0**	43.1	< 0.001
Cefepime	40 (80.0)	18 (36.0)	**44.0**	40.0	< 0.001
Imipenem	15 (30.0)	13 (26.0)	**4.0**	13.9	NS
Meropenem	14 (28.0)	12 (24.0)	**4.0**	11.5	NS
Gentamicin	35 (70.0)	21 (42.0)	**28.0**	40.0	NS
Amikacin	22 (44.0)	15 (30.0)	**14.0**	20.8	NS
Ciprofloxacin	44 (88.0)	21 (42.0)	**46.0**	52.2	< 0.001
Levofloxacin	40 (80.0)	22 (44.0)	**36.0**	45.0	< 0.05
Tetracycline	47 (94.0)	29 (58.0)	**36.0**	38.5	< 0.001
Trimethoprim–sulfamethoxazole	39 (78.0)	21 (42.0)	**36.0**	46.2	< 0.05

*Note:* Statistical test, McNemar′s test for paired binary outcomes (pre‐ vs. postcuring within the same isolate); two‐sided *p* values reported. Absolute change (*%*) = precuring resistant*%* − postcuring resistant*%*.Reduction rate (*%*) = (precuring resistant − postcuring resistant)/precuring resistant × 100. 95% CIs for paired‐difference and Bayesian sensitivity analyses are reported in the Statistical Analysis section. Macrolides were not included in this analysis owing to intrinsic *E. coli* resistance.

^∗^Statistically significant at *p* ≤ 0.05.

Abbreviation: NS, nonsignificant.

**Table 4:** Virulence traits before and after SDS‐induced plasmid curing in *E. coli* isolates (n = 50).

Virulence factor	Precuring, *n* (%)	Postcuring, *n* (%)	Reduction rate (%)	*p*
Hemolysin production	30 (60.0)	14 (28.0)	53.3	< 0.001 ^∗^
Motility	43 (86.0)	16 (32.0)	62.8	< 0.001 ^∗^

*Note:* Statistical test, McNemar′s test for paired binary outcomes (pre‐ vs. postcuring within the same isolate). Hemolysin production assessed on 5% sheep blood agar (*β*‐hemolysis); motility assessed on 0.3% semisolid motility medium. 95% CIs for paired‐difference are reported in the Statistical Analysis section.

^∗^Statistically significant at *p* ≤ 0.05.

**Table 5:** Integrated molecular–phenotypic outcomes following plasmid curing in multidrug‐resistant (MDR) *E. coli* isolates (n = 50).

Phenotypic–molecular subgroup	*n*	%	Plasmid loss	MDR	Hemolysin	Motility	Interpretation
Complete plasmid loss with full phenotypic reversion	39	78.0	Complete	Lost	Lost	Lost	Strong evidence of plasmid‐associated MDR and virulence
Complete plasmid loss with partial phenotypic reversion (subtotal)	3	6.0	Complete	Mixed	Mixed	Mixed	Incomplete phenotypic response despite complete plasmid elimination
└ MDR loss only	1	2.0	Complete	Lost	Retained	Retained	MDR plasmid‐associated; virulence chromosomal
└ Virulence loss only (hemolysin and motility)	1	2.0	Complete	Retained	Lost	Lost	Virulence plasmid‐associated; MDR chromosomal
└ MDR and hemolysin loss with motility retained	1	2.0	Complete	Lost	Lost	Retained	Motility independently regulated/chromosomal
Partial plasmid loss with partial phenotypic reversion	5	10.0	Partial	Reduced	Reduced	Reduced	Mixed plasmid–chromosomal genetic control
No plasmid loss with no phenotypic reversion	3	6.0	None	Retained	Retained	Retained	Chromosomal resistance or highly stable plasmids
**Overall molecular–phenotypic association**	Spearman^′^s *r*_*S*_ = 0.83, *p* < 0.001 (Bayesian sensitivity analysis with noninformative Beta(1,1) priors yielded posterior probability > 0.99 of true positive association).						

*Note:* Percentages calculated based on the total number of isolates (*n* = 50). Molecular outcomes were coded as ordinal variables (complete plasmid loss = 2; partial plasmid loss = 1; no plasmid loss = 0); phenotypic outcomes were coded as ordinal variables (complete phenotypic reversion = 2; partial reversion = 1; no reversion = 0). The use of Spearman′s rank correlation (rather than a simple categorical association test) was therefore appropriate for these ordinal variables; the result was independently confirmed by a Cochran–Armitage trend test (*p* < 0.001). “Mixed” denotes selective, nonuniform loss or retention of antimicrobial resistance and/or virulence traits following plasmid curing. MDR conclusions are population‐level and consistent with but not direct genotypic confirmation of plasmid‐mediated resistance and virulence.

## 3. Results

### 3.1. Sample Collection, Identification, and Integrated MDR Analysis

**Figure 1:**
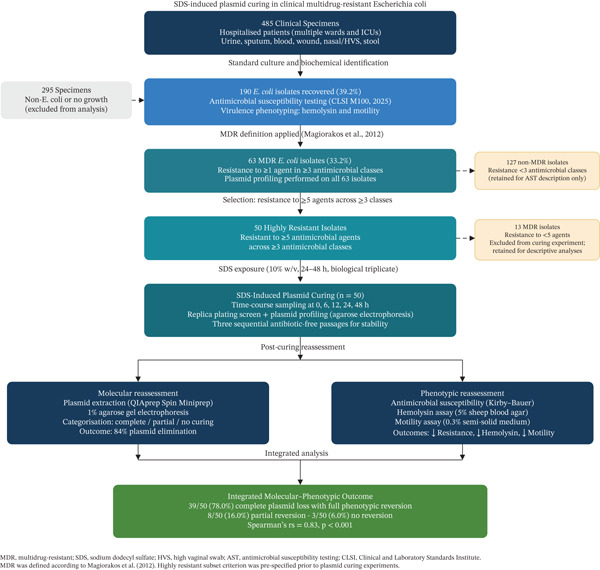
Study flow diagram. Of the 485 clinical specimens collected, 190 *E. coli* isolates were recovered (39.2%). Antimicrobial susceptibility testing and virulence phenotyping were performed for all 190 isolates. Of these, 63 (33.2%) were classified as MDR according to the [14] definition. SDS‐induced plasmid curing and postcuring molecular and phenotypic reassessment were applied to the highly resistant subset (*n* = 50; resistance to ≥ 5 antimicrobial agents across ≥ 3 antimicrobial classes). The remaining 13 MDR isolates resistant to < 5 agents were excluded from curing but retained in descriptive analyses.

From the 485 clinical specimens collected, 190 *E. coli* isolates (39.2%) were recovered (Figure 1, study flow diagram). Subsequent analyses focused on these 190 isolates and their downstream MDR subset. The highest absolute number of MDR isolates was derived from urine samples (160 specimens), with 72 isolates (45.0%) confirmed as *E. coli*. Sputum specimens contributed 35 isolates (43.8% of those collected), exhibiting the highest resistance intensity, with a mean MARI of 0.71 (Table 6). Blood, wound swabs, nasal and high vaginal swabs, and stool samples contributed 23, 31, 14, and 15 isolates, respectively, with MDR prevalence ranging from 26.7% to 37.5% (Table 6).

**Table 6:** Integrated *E. coli* isolation, MDR burden, and resistance intensity by sample type.

Sample type	Total specimens (*N*)	*coli* isolates, *n* (%)	MDR isolates, *n* (%)	Mean MARI (resistance intensity)
**Urine (including catheters)**	160	72 (45.0)	27 (37.5)	0.69
**Sputum**	80	35 (43.8)	12 (34.3)	0.71
**Blood**	55	23 (41.8)	7 (30.4)	0.55
**Wound swabs**	85	31 (36.5)	9 (29.0)	0.62
**Nasal and high vaginal swabs**	50	14 (28.0)	4 (28.6)	0.58
**Stool**	55	15 (27.3)	4 (26.7)	0.54
**Total**	**485**	**190 (39.2)**	**63 (33.2)**	**0.63**

*Note:* Calculated per isolate as the number of antibiotics to which the isolate was resistant divided by the total number of antibiotics tested (mean values reported per sample type). MDR was defined according to [14]. Macrolides were excluded from MARI/MDRI calculations because of intrinsic *E. coli* resistance.

Abbreviation: MARI, multiple antibiotic resistance index.

### 3.2. MDR Prevalence and Resistance Distribution by Hospital Ward

Among the 190 *E. coli* isolates, 63 (33.2%) were classified as MDR according to Magiorakos et al. [14]. The average MARI in the MDR subset was 0.63, indicating high resistance intensity, whereas the MDRI (reflecting class‐level resistance complexity) averaged 0.48 in the MDR subset.

The prevalence and intensity of MDR *E. coli* vary significantly across hospital wards. The ICU consistently exhibited the highest resistance intensity (mean = 0.78) and complexity (mean = 0.55). Medical and surgical wards followed with a mean MDRI of (0.45 and 0.47, respectively), whereas pediatric and obstetrics and gynecology wards showed comparatively lower, but still clinically relevant, prevalence. (Table S1).

### 3.3. Antibiotic Resistance and Virulence Profiles

#### 3.3.1. Antibiotic Resistance and Coresistance Among the total Isolated *E. coli* (*n* = 190)

Antimicrobial susceptibility testing of the full *E. coli* cohort (*n* = 190) revealed a substantial resistance burden across multiple antimicrobial classes (Table S2). Across the entire 190‐isolate *E. coli* cohort, The highest resistance rates were observed for commonly used agents, including ampicillin (40.5%), ceftriaxone (40.0%), and ciprofloxacin (38.9%), reflecting global trends of increasing resistance to *β*‐lactams, third‐generation cephalosporins, and fluoroquinolones. Resistance was also prominent for amoxicillin–clavulanate and levofloxacin (36.3% each), tetracycline (34.2%), and trimethoprim–sulfamethoxazole (31.6%), indicating widespread antimicrobial exposure and selection pressure. Although carbapenem resistance remained comparatively low (imipenem 11.6%, meropenem 11.1%), its presence is clinically concerning. The unusually high nitrofurantoin resistance among urinary isolates (52.8%) likely reflects the local prescribing pattern at our tertiary care institution, where nitrofurantoin is the first‐line empirical agent for both community‐onset and recurrent UTIs and is widely available without prescription in the regional pharmacy network, generating sustained selective pressure consistent with previous Egyptian and regional reports [19, 20].

#### 3.3.2. Antibiotic Resistance and Coresistance in MDR Isolates (*n* = 63)

In contrast, when restricted to the MDR subset (*n* = 63), resistance rates were substantially higher across all antimicrobial classes, as detailed in Table 2. Resistance to ampicillin and amoxicillin–clavulanate was 90.5% and 85.7%, respectively. High resistance to third‐generation cephalosporins was observed, with ceftriaxone and ceftazidime resistance rates of 88.9% and 82.5%, respectively. Fluoroquinolone resistance was also frequent, with resistance rates of 84.0% for ciprofloxacin and 79.4% for levofloxacin. Carbapenem susceptibility remained relatively high, with 77.8% of isolates susceptible to meropenem and 76.2% to imipenem. Among urinary MDR isolates, nitrofurantoin resistance was 62.9%. Throughout the manuscript, percentages stated for the 190 *E. coli* cohort and the 63‐isolate MDR subset are reported separately and clearly labeled to avoid confusion between the two populations.

### 3.4. MDR Profiles

Analysis of MDR patterns among *E. coli* isolates (*n* = 63) revealed diverse resistance profiles (Table 1). The most prevalent MDR profile, involving resistance to *β*‐lactams, cephalosporins, fluoroquinolones, lincosamides, and phenicols (with erythromycin retained only as a phenotypic profile descriptor; see Methods), was identified in 12 isolates (19.0%) with an MDRI of 0.70. Additional frequent profiles, including CIP–LBC–ERY–P–CD–OFX and LBC–P–GX–CN–OFX–AZN–ERY–CRO, were observed in 10 (15.9%) and 8 (12.7%) isolates, respectively. Extended *β*‐lactam resistance profiles involving third‐generation cephalosporins and carbapenems were detected in seven isolates (11.1%). Fifteen isolates (23.8%) exhibited less complex MDR profiles with MDRI values ranging from 0.30 to 0.60.

### 3.5. Virulence Profiles

A high cocarriage of virulence‐associated phenotypes was observed among the *MDR E. coli* (*n* = 63) isolates. Motility was detected in 85.7% of isolates, highlighting its role as a key factor in bacterial colonization and dissemination, whereas hemolysin production was present in 61.9%, reflecting an enhanced capacity for tissue invasion and host damage. The frequent coexistence of these virulence traits with MDR underscores the pathogenic potential of MDR *E. coli* strains and suggests possible, but not yet genetically demonstrated, colocalization between resistance and virulence determinants on shared genetic platforms.

### 3.6. Phenotypic Resistance Reversion After SDS‐Induced Plasmid Curing

Following SDS‐induced plasmid curing, a marked and statistically significant reduction in AMR was observed across most antibiotic classes (Table 3). The most pronounced effects were detected among *β*‐lactams and fluoroquinolones. Resistance to ampicillin decreased from 92.0% (46/50) precuring to 42.0% (21/50) postcuring, representing an absolute reduction of 50.0 percentage points and a relative reduction of 54.3% (*p* < 0.001; 95% CI for paired‐difference: 35.4%–62.9%). Similar patterns were observed for ceftriaxone (absolute reduction: 46.0%; relative reduction: 51.1%) and ciprofloxacin (absolute reduction: 46.0%; relative reduction: 52.2%), reductions consistent with plasmid‐mediated resistance loss, although direct gene‐level evidence (e.g., loss of bla_CTX-M_, bla_TEM_, bla_SHV_, or qnr *genes*) was not obtained in the present work and is acknowledged as a limitation.

Moderate but significant reductions were also documented for amoxicillin–clavulanate (46.5%), ceftazidime (43.1%), cefepime (40.0%), piperacillin–tazobactam (40.0%), levofloxacin (45.0%), tetracycline (38.5%), and trimethoprim–sulfamethoxazole (46.2%). In contrast, resistance to carbapenems showed minimal, nonsignificant changes following curing (imipenem: 13.9%; meropenem: 11.5%), suggesting predominantly chromosomal or highly stable resistance mechanisms, such as porin loss (OmpF/OmpC downregulation), upregulation of efflux pumps (e.g., AcrAB‐TolC), and chromosomally encoded carbapenemases or ampC hyperproduction, which are not eliminated by SDS‐mediated plasmid curing. Overall, these findings indicate that a substantial fraction of phenotypic resistance in this MDR *E. coli* cohort was reversible following SDS exposure, consistent with but not directly demonstrative of plasmid‐borne resistance determinants. Phenotypic reversion alone cannot definitively confirm plasmid‐mediated resistance in the absence of pre/post curing genotypic data, and the present conclusions are interpreted accordingly.

### 3.7. Virulence Phenotype Reduction

Plasmid curing resulted in a significant attenuation of virulence‐associated phenotypes among *E. coli* isolates (*n* = 50) (Table 4). Hemolysin production decreased from 60.0% (30/50) precuring to 28.0% (14/50) postcuring, representing a 53.3% reduction (McNemar′s test, *p* < 0.001; 95% CI for paired‐difference: 18.6%–43.4%). Similarly, bacterial motility was markedly reduced, declining from 86.0% (43/50) in the parental strains to 32.0% (16/50) postcuring, corresponding to a 62.8% decrease (*p* < 0.001; 95% CI: 38.4–67.2%). These results indicate that plasmid elimination significantly impacts key virulence traits, supporting the contribution of plasmid‐borne determinants to these virulence‐associated phenotypes, while acknowledging that hemolysin and motility may also be chromosomally encoded in a subset of strains.

### 3.8. Time‐Course of Curing

Kinetic analysis of plasmid curing among *E. coli* isolates (*n* = 50) demonstrated rapid and progressive plasmid elimination over time (Table 7). Plasmid retention declined sharply from 100% at baseline to 59.7% at 6 h, 35.0% at 12 h, and 12.3% at 24 h, reaching 1.9% after 48 h. This marked reduction in plasmid carriage was accompanied by a parallel decrease in AMR, with resistant isolates falling from 100% (50/50) at baseline to 4% (2/50) at 48 h, corresponding to an overall 96% reduction in resistance.

**Table 7:** Time‐course dynamics of SDS‐induced plasmid curing.

Time (h)	Plasmid retention (%)	Resistant isolates, *n* (%)	Resistance reduction (%)	Reduction rate (%/h)
0	100	50 (100)	0	—
6	59.7	33 (66)	34	5.67
12	35.0	24 (48)	52	3.00
24	12.3	8 (16)	84	2.67
48	1.9	2 (4)	96	0.50

*Note:* SDS, sodium dodecyl sulfate (10% *w*/*v* final). Plasmid retention is the percentage of isolates retaining detectable plasmid bands by agarose gel electrophoresis at each time point relative to the total tested (*n* = 50). Resistance reduction (%) is the percentage decrease in resistant isolates relative to baseline (0 h). Reduction rate (%/h) is the resistance reduction divided by the time interval. All time‐course experiments were performed in biological triplicate with technical duplicates per time point.

The highest rate of resistance reduction occurred during the early phase of exposure, particularly within the first 6–12 h (5.67% and 3.00% per hour, respectively), followed by a gradual decline in the reduction rate at later time points. This kinetic pattern is consistent with early disruption of plasmid replication and segregation, with a small subset of more stable plasmids persisting during prolonged exposure, as previously described in the SDS‐curing literature; we did not directly measure replication or segregation processes in this study, and the mechanistic explanation is therefore offered as a literature‐based interpretation rather than a direct experimental finding.

### 3.9. Plasmid Profile of MDR Clinical Isolates of *E. coli* Pre‐ and Postcuring

**Figure 2:**
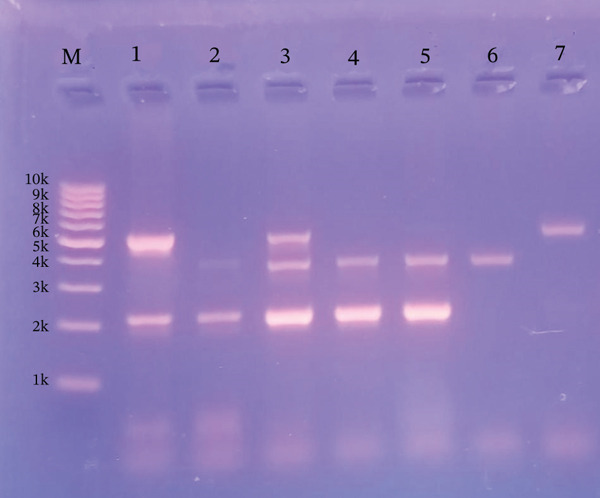
Agarose gel electrophoresis of plasmid profiles from selected MDR *E. coli* isolates prior to SDS curing. M: 10‐kbp ladder; Lanes 2, 4, and 5: two plasmid bands at 2 and 4 kbp; Lane 1: two bands at 2 and 5 kbp; Lane 3: three bands at 2, 4, and 5 kbp; Lanes 6 and 7: one band at 4 and 5 kbp.

**Figure 3:**
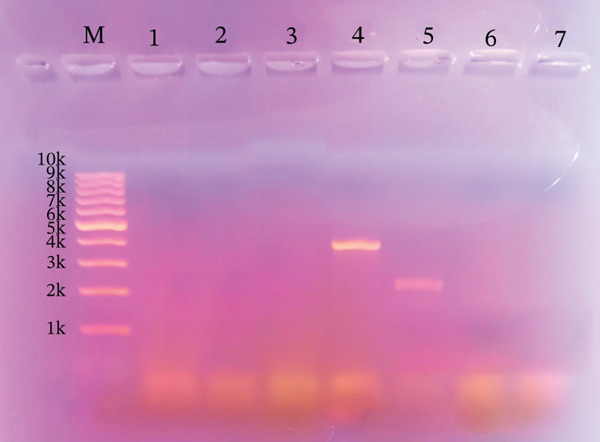
Agarose gel electrophoresis of plasmid profiles of the same selected MDR *E. coli* isolates post‐SDS curing. Lanes 1, 2, 3, 6, and 7: complete loss of plasmid bands; Lanes 4 and 5: loss of one band at 2 and 4 kbp, respectively.

A total of 50 MDR isolates were screened for plasmids. Within the resolvable range of the 1–10‐kb ladder used, all selected MDR (*n* = 50) isolates harbored a plasmid with a size range of 1.5–5 kb. The plasmid profiles found among MDR tested isolates included 1–3 bands of different sizes (Figure 2). A single plasmid band was found among 23 isolates, with sizes ranging from 1.5 to 5 kbp; two plasmid bands were found in 16 isolates of sizes ranging from 2 to 4 kbp; three plasmid bands were found in 11 isolates of sizes ranging from 2 to 5 kbp. Plasmid curing procedure was carried out, and the plasmid bands were not seen after electrophoretic separation in 42 isolates (84%) of SDS‐treated isolates classified as 21 isolates among isolates with one band, 13 isolates among isolates with 2 bands, 8 isolates among isolates with 3 bands, whereas the number of bands decreased in 5 isolates including 2 isolates among isolates with 2 bands (one band loss) and 3 isolates among isolates with 3 bands (2 bands loss) and no loss of bands was observed in 3 isolates including 2 isolates among isolates with one band and 1 isolate among isolates with 2 bands (Table 8, Figure 3).

**Table 8:** Plasmid profile of MDR clinical isolates of *E. coli* pre‐ and post‐SDS curing.

*N*. of isolates (*t* *o* *t* *a* *l* = 50)	*N*. Of plasmid bands	Plasmid size (kbp)	Postcuring loss of plasmid bands		
			Complete loss (*n* = 42)	Partial loss (*n* = 5)	No loss (*n* = 3)
23	1	1.5–5	21	—	2
16	2	2–4	13	2 (one band loss)	1
11	3	2–5	8	3 (2 bands loss)	—

*Note:* Plasmid sizes were estimated by comparison with a 1–10‐kb GelPilot ladder (Qiagen, Germany); plasmids outside this resolvable range cannot be excluded. Partial loss refers to a reduction in the number of detectable plasmid bands following SDS curing. Complete loss is defined as the disappearance of all detectable plasmid bands compared with the parental isolate.

### 3.10. Molecular and Phenotypic Outcomes of Plasmid Curing

Integrated analysis of molecular plasmid profiles and corresponding phenotypic changes following SDS‐induced curing revealed a strong concordance between plasmid elimination and reversal of AMR and virulence traits among *MDR E. coli* isolates (Table 5). Complete plasmid loss was confirmed in 42/50 isolates (84.0%), of which the majority (39 isolates, 78.0%) exhibited complete phenotypic reversion characterized by simultaneous loss of MDR, hemolysin production, and motility. This finding is consistent with plasmid‐mediated determinants for both resistance and virulence in the majority of these isolates, although direct gene‐level confirmation was not performed.

Among isolates demonstrating complete plasmid elimination, three (6.0%) showed partial phenotypic reversion, reflecting heterogeneous genetic architectures. Within this subgroup, one isolate exhibited loss of MDR only while retaining virulence traits, suggesting plasmid‐mediated resistance with chromosomally encoded virulence determinants. Conversely, one isolate retained MDR but lost both hemolysin production and motility, indicating plasmid‐associated virulence with chromosomal resistance mechanisms. A third isolate demonstrated concurrent loss of MDR and hemolysin while retaining motility, supporting the notion that motility may be independently regulated or chromosomally encoded in certain strains.

Partial plasmid loss was detected in five isolates (10.0%), all of which displayed partial phenotypic reversion manifested as reduced AMR and attenuated virulence traits. This pattern is consistent with mixed plasmid–chromosomal genetic control and incomplete elimination of resistance and virulence‐associated plasmid elements. In contrast, three isolates (6.0%) showed no detectable plasmid loss following SDS exposure and correspondingly retained their MDR phenotype and virulence traits, suggesting chromosomal resistance mechanisms or the presence of highly stable plasmids refractory to curing.

When analyzed as summary categories, persistence of MDR was observed in 11 isolates, while persistence of virulence traits was documented in nine isolates, underscoring the heterogeneity of resistance and virulence architectures among clinical *E. coli* strains. Both molecular and phenotypic outcomes were coded as ordinal variables (no loss = 0; partial loss = 1; complete loss = 2), and Spearman′s rank correlation was therefore used as a rank‐based association test for ordinal data, with the result independently confirmed by a Cochran–Armitage trend test (*p* < 0.001), justifying the use of correlation rather than a simple categorical association test. Overall, a strong positive rank‐based association was identified between plasmid curing status and phenotypic reversion (Spearman^′^s *r*_*S*_ = 0.83, *p* < 0.001; Bayesian sensitivity analysis with noninformative Beta(1,1) priors yielded a posterior probability > 0.99 of true positive association), supporting plasmids as the predominant though not exclusive drivers of MDR and virulence in the majority of the studied isolates.

## 4. Discussion

MDR in infectious diseases poses a serious threat to effective treatment, making the understanding of resistance patterns essential for controlling bacterial drug resistance [18]. Accordingly, this study investigated plasmid profiling of MDR *E. coli* isolated from diverse clinical specimens, with MDR isolates confirmed prior to plasmid extraction, analysis, and SDS‐mediated curing.

Analysis of 485 clinical specimens from multiple hospital wards identified *E. coli* as the predominant pathogen, with an isolation rate of 39.2% (190/485), highlighting its etiological importance in nosocomial infections. This finding is consistent with reports by Al Qahtani et al. [21], who described *E. coli* as a major pathogen across various clinical sources. Of the 190 *E. coli* isolates, 63 (33.2%) were MDR, a proportion closely comparable to that reported by Thapa Shrestha et al. [22], who documented MDR in 33.53% of urinary *E. coli* isolates. Urine samples yielded the highest *E. coli* recovery (45.0%), with sputum isolates exhibiting the highest mean MARI (0.71); detailed comparisons with regional cohorts and the consistency of the present MDR prevalence with international tertiary‐care benchmarks [19, 23] are summarized here without restating the figures already provided in the Results.

Within the MDR subset (*n* = 63), resistance to *β*‐lactams and fluoroquinolones was pronounced and consistent with the dissemination of ESBL‐associated plasmids and colocated fluoroquinolone resistance determinants [16, 24]. The high ceftriaxone resistance is suggestive of, but does not confirm, ESBL carriage; phenotypic ESBL confirmation (e.g., the CLSI M100 cefotaxime/ceftazidime ± clavulanic acid double − disk synergy test) and molecular detection of bla_CTX-M_, bla_TEM_, and bla_SHV_ genes were not performed in the present work and are explicitly acknowledged as a limitation. Statements regarding ESBL‐mediated plasmid resistance in this manuscript should therefore be interpreted as inferential and require future genotypic confirmation. In contrast, carbapenems retained relatively preserved activity, with susceptibilities of 77.8% to meropenem and 76.2% to imipenem, supporting an ESBL‐driven MDR profile rather than widespread carbapenemase dissemination [24–26]. Resistance levels exceeded those reported in some secondary‐care settings and are consistent with sustained clinical antimicrobial exposure in tertiary‐care environments [19, 27–30].

Among urinary MDR isolates (*n* = 27), nitrofurantoin resistance reached 62.9%, substantially exceeding rates reported in non‐ICU uropathogenic cohorts, including the 13.53% documented by Thapa Shrestha et al. [22]. This elevated resistance likely reflects recurrent infection, catheterization, repeated empiric therapy, and the local availability of nitrofurantoin without prescription within the regional pharmacy network [1, 2].

Globally, the rising burden of MDR gram‐negative bacteria, particularly ESBL‐ and carbapenem‐resistant *E. coli*, constitutes a major public health threat and is associated with increasing resistance‐related mortality worldwide [31, 32]. The MDR prevalence observed in the present study is consistent with reports from tertiary‐care hospitals across the Middle East and Africa [33–35]. Regional variability in MDR has been linked to differences in antibiotic utilization patterns, healthcare infrastructure, and stewardship implementation [35–41].

A high prevalence of virulence‐associated phenotypes was detected among MDR *E. coli* prior to plasmid curing (motility: 85.7%, hemolysin: 61.9%), exceeding rates reported in several MDR uropathogenic cohorts but consistent with recent Egyptian data [22, 30, 42, 43]. The elevated virulence phenotype burden supports evidence that *E. coli* virulence determinants are frequently plasmid‐associated and colocalized with AMR genes, facilitating coselection under sustained antimicrobial pressure [6, 44] and promoting plasmid persistence despite fitness costs [45].

Following exposure to SDS, a pronounced reversal of phenotypic AMR was observed across multiple antibiotic classes, with the most significant reductions occurring among *β*‐lactams and fluoroquinolones, an observation that is consistent with a plasmid‐mediated basis for resistance and the known colocalization of ESBL genes and plasmid‐mediated quinolone resistance determinants on transferable mobile genetic elements [8]. The magnitude of resistance reversion observed exceeded that reported in some SDS curing studies, likely reflecting differences in resistance architecture, as isolates with higher plasmid burdens exhibited greater susceptibility restoration. Moderate reductions across additional antimicrobial classes support the presence of clustered plasmid‐linked resistance determinants enabling simultaneous loss after plasmid elimination, whereas minimal or nonsignificant changes in aminoglycoside and carbapenem resistance indicate heterogeneous mechanisms with chromosomal contributions, including porin alterations (OmpF/OmpC), efflux pump upregulation (AcrAB–TolC), and chromosomal carbapenemase or ampC hyperproduction, which are not removed by plasmid curing [6, 24, 26].

Plasmid curing significantly attenuated virulence‐associated phenotypes, with hemolysin production declining from 60.0% to 28.0% and motility from 86.0% to 32.0%, consistent with normalization toward population baselines reported in broader clinical populations (e.g., a pooled hlyA prevalence of 22.1% [46], and hemolysin positivity of approximately 50.4% [43]. The concurrent attenuation of resistance and virulence supports plasmid coselection theory, whereby antimicrobial pressure maintains plasmids carrying multiple adaptive traits and plasmid loss results in simultaneous phenotypic reduction [6, 45]. Residual virulence in a subset of cured isolates suggests heterogeneous genetic architectures, with some virulence determinants likely chromosomally encoded [44]. Importantly, hemolysin and motility are not always plasmid‐encoded; chromosomal hlyA loci and flagellar (fliC/flhDC) regulons are well documented in *E. coli*, and the present interpretation accordingly emphasizes a population‐level rather than universal plasmid contribution.

In the present study, all MDR *E. coli* isolates harbored plasmids ranging in detectable size from 1.5 to 5 kb within the resolvable range of the 1–10‐kb ladder used, with plasmid profiles consisting of one to three bands. Following SDS curing, plasmid bands were eliminated in 42 isolates (84%), indicating a high curing efficiency, with the explicit caveat that larger ESBL‐/carbapenemase‐associated plasmids (typically 50–250 kb) lie outside the resolvable range of the ladder employed and therefore cannot be excluded; the plasmid size distribution reported here should not be overgeneralized. This finding closely resembles reports by Sulaiman et al. [47], who achieved complete plasmid elimination in 100% of isolates with significant MAR indices. Similarly, Aladejana et al. [17] reported plasmid carriage in all except one isolate, with residual plasmid bands of reduced molecular weight persisting after curing. Comparable findings were reported by Alkali et al. [48], who observed plasmid carriage in 90% of MDR isolates, with plasmid sizes ranging from 6.0 to 20 kb. The plasmid sizes observed in the present study align with earlier reports of small plasmids (< 2.1 kb [49]) and 1.2–5.3 kb [50], although they contrast with a study reporting larger plasmids (6.0–20 kb; Alkali et al. [48]).

Time‐course analysis of SDS‐induced plasmid curing demonstrated a biphasic kinetic pattern, with rapid early loss followed by persistence of a small stable subpopulation, consistent with classical SDS curing studies [8, 51–53]. The declining rate of resistance loss supports a two‐phase model involving early displacement of less stable plasmids and a slower terminal phase dominated by stable plasmid–host combinations [45, 54], although direct evidence for plasmid replication or segregation disruption was not generated in the present study, and these mechanistic interpretations are presented as literature‐based rather than as direct findings.

Molecular analysis demonstrated strong concordance between plasmid loss and phenotypic reversion following SDS curing. Plasmid elimination was confirmed in 42/50 isolates (84.0%), of which 39 (78.0%) showed full susceptibility restoration, 8 (16.0%) showed partial reversion, and only 3 (6.0%) exhibited no phenotypic change, yielding a strong molecular–phenotypic correlation (*r*_*s*_ = 0.83). The presence of partial and nonreverting isolates is biologically expected and reflects mixed resistance architectures involving plasmid‐borne genes, chromosomal mutations, and highly stable plasmids [4, 55].

Finally, although SDS‐induced plasmid curing efficiently reverses MDR and virulence phenotypes in vitro, SDS itself is not clinically applicable as a therapeutic intervention because of its nonspecific cytotoxic, surfactant, and proinflammatory properties. The present results should therefore be interpreted as defining plasmid curing as an experimental laboratory tool for dissecting plasmid‐mediated resistance and virulence, and as a conceptual framework to inform future development of more selective antiplasmid strategies (e.g., CRISPR–Cas conjugative delivery, antiplasmid small molecules, or fitness‐based plasmid displacement)—none of which were tested here. Clinical or in vivo applicability of plasmid curing as such remains hypothetical and beyond the scope of this study.

## 5. Conclusion

Our study demonstrates that SDS is an effective experimental plasmid‐curing agent, leading to a pronounced reduction in both AMR and virulence traits in clinical MDR *E. coli*. The strong molecular–phenotypic concordance supports plasmids as predominant though not exclusive drivers of MDR and virulence in most isolates, whereas residual resistance reflects chromosomal or highly stable plasmid determinants. SDS‐induced plasmid curing should be regarded as a valuable laboratory tool to dissect plasmid‐mediated resistance mechanisms rather than as a clinically translatable therapy, and its findings can inform plasmid‐targeted research strategies aligned with antimicrobial stewardship in hospital settings.

## 6. Study Limitations

• Molecular characterization of specific resistance and virulence genes was not performed; however, phenotypic plasmid curing was used as the primary endpoint, with strong molecular–phenotypic concordance supporting the findings.

• Plasmid size determination was limited to the 1–10‐kb range of the molecular‐weight ladder, excluding larger clinically relevant plasmids, although resistance reversion results remain unaffected.

• Plasmid incompatibility (Inc) typing and whole‐plasmid sequencing were not undertaken, as gel‐based confirmation of plasmid loss is an accepted standard for assessing curing efficacy.

• The work was conducted entirely in vitro; SDS is not clinically applicable due to cytotoxicity, and the study is intended as a laboratory investigation of plasmid‐mediated resistance and virulence rather than a therapeutic approach.

**Nomenclature**
MDR: multidrug resistance
MARI: multiple antibiotic resistance index
MDRI: multidrug resistance index
SDS: sodium dodecyl sulfate
ESBL: extended‐spectrum *β*‐lactamase
ICU: intensive care unit
ARG: antibiotic resistance gene
CLSI: Clinical and Laboratory Standards Institute
CI: confidence interval
LB: Luria–Bertani
PCR: polymerase chain reaction

## Author Contributions

Alyaa I. Eliwa contributed to the conceptualization, methodology, data curation, and formal analysis. Hagar S.M. Nassar contributed to laboratory investigations, data acquisition, and validation. Maha M. Eldahshan contributed to supervision, interpretation of results, and critical revision of the manuscript.

## Funding

No funding was received for this manuscript.

## Disclosure

All authors read and approved the final manuscript.

## Ethics Statement

The study was approved by the Institutional Ethics Committee of the Faculty of Medicine, Menoufia University (IRB Reference Number: 2026CPATH12). All procedures were conducted in accordance with the Declaration of Helsinki and relevant institutional guidelines.

## Conflicts of Interest

The authors declare no conflicts of interest.

## Supporting information


**Supporting Information** Additional supporting information can be found online in the Supporting Information section. Table S1: MDR distribution, intensity (MARI), and complexity (MDRI) by hospital ward. Table S2: Antibiotic resistance and coresistance in isolated *E. coli* (*n* = 190).

## Data Availability

The datasets generated and/or analyzed during the current study are available from the corresponding author upon reasonable request.
