# Supplementary material for: Disarming Multidrug‐Resistant Pathogens: Sodium Dodecyl Sulfate–Induced Plasmid Curing for the Reversal of Resistance and Virulence in Escherichia coli
Source: Int J Microbiol. 2026 Jun 19;2026:5568321. doi: 10.1155/ijm/5568321 (PMC13280985; doi:10.1155/ijm/5568321)
Supplement: Supplementary file 1 — Supporting Information Additional supporting information can be found online in the Supporting Information section. Table S1: MDR distribution, intensity (MARI), and complexity (MDRI) by hospital ward. Table S2: Antibiotic resistance and coresistance in isolated E. coli (n = 190). [file IJM-2026-5568321-s001.docx]

**Supplementary Tables**

**Supplementary Table 1. MDR Distribution, Intensity (MARI), and Complexity (MDRI) by Hospital Ward**

| **Hospital Ward** | **Total Isolates (A)** | **MDR Isolates (B)** | **MDR Prevalence (%) (B/A)** | **Mean MARI** | **Mean MDRI** | **Interpretation** |
| --- | --- | --- | --- | --- | --- | --- |
| **Intensive Care Unit (ICU)** | **40** | **14** | **35.0** | **0.78** | **0.55** | **Highest resistance intensity and complexity.** |
| **Medical Wards** | **50** | **18** | **36.0** | **0.61** | **0.45** | **Highest MDR prevalence within patient group.** |
| **Surgical Wards** | **45** | **14** | **31.1** | **0.65** | **0.47** | **Reflects intensive antibiotic use pressure.** |
| **Pediatrics** | **30** | **9** | **30.0** | **0.51** | **0.37** | **Lowest resistance intensity and complexity.** |
| **Obstetrics & Gynecology** | **25** | **8** | **32.0** | **0.58** | **0.42** | **Moderate MDR prevalence; important clinical ward.** |
| **Total** | **190** | **63** | **33.2** | **0.63** | **0.48** | **Institutional MDR burden benchmark.** |

Footnotes: MDR was defined as acquired non-susceptibility to at least one agent in three or more antimicrobial categories, in accordance with Magiorakos et al. (2012) **[14]**. MARI (Multiple Antibiotic Resistance Index) = number of antibiotics to which the isolate was resistant / total number of antibiotics tested (mean per ward). MDRI (Multidrug Resistance Index) = number of antimicrobial classes to which the isolate was resistant / total number of antimicrobial classes tested. Macrolides were excluded from MARI/MDRI calculations because of intrinsic *E. coli* resistance. Susceptibility interpreted per CLSI M100 (2025).

**Supplementary Table 2. Antibiotic Resistance and Co-Resistance in Isolated *E.coli* (n=190):**

| **Antibiotic Class** | **Antibiotic** | **Resistant (N)** | **Resistance (%)** | **Co-Resistance Rate (%)** |
| --- | --- | --- | --- | --- |
| **β-Lactams** | **Ampicillin (AMP)** | **77** | **40.5** | **40.5 (≥1 β-Lactam)** |
|  | **Amoxicillin-clavulanate (AMC)** | **69** | **36.3** | **—** |
|  | **Piperacillin-tazobactam (TZP)** | **52** | **27.4** | **—** |
| **Cephalosporins** | **Ceftriaxone (CRO)** | **76** | **40.0** | **40.0 (≥1 3rd Gen Cephalosporin)** |
|  | **Ceftazidime (CAZ)** | **68** | **35.8** | **—** |
|  | **Cefepime (FEP)** | **62** | **32.6** | **—** |
| **Carbapenems** | **Imipenem (IPM)** | **22** | **11.6** | **11.6 (≥1 Carbapenem)** |
|  | **Meropenem (MEM)** | **21** | **11.1** | **—** |
| **Aminoglycosides** | **Gentamicin (GEN)** | **56** | **29.5** | **—** |
|  | **Amikacin (AK)** | **36** | **18.9** | **—** |
| **Fluoroquinolones** | **Ciprofloxacin (CIP)** | **74** | **38.9** | **38.9 (≥1 Fluoroquinolone)** |
|  | **Levofloxacin (LEV)** | **69** | **36.3** | **—** |
| **Others** | **Tetracycline (TE)** | **65** | **34.2** | **—** |
|  | **Trimethoprim-sulfamethoxazole (SXT)** | **60** | **31.6** | **—** |
|  | **Nitrofurantoin* (urine isolates, n=72)** | **38** | **52.8** | **—** |

Footnotes: Resistance percentages are calculated against the full *E. coli* cohort (n = 190), distinct from the higher resistance rates observed in the MDR subset (n = 63; Table 2 of the main manuscript). *Nitrofurantoin susceptibility was tested only for urinary isolates (n = 72) in accordance with its clinical indication. Macrolides were not tested because of intrinsic *E. coli* resistance, which would artefactually inflate resistance indices and obscure plasmid-mediated post-curing changes; erythromycin appearing in MDR profile descriptors (main Table 3) is included as a phenotypic descriptor only and was not used in MARI or MDRI calculations. Susceptibility interpreted per CLSI M100 (2025); MDR defined per Magiorakos et al. (2012) **[14]**.
